# Experimental and Numerical Analysis of High-Resolution Injection Technique for Capillary Electrophoresis Microchip

**DOI:** 10.3390/ijms12063594

**Published:** 2011-06-03

**Authors:** Chin-Lung Chang, Jik-Chang Leong, Ting-Fu Hong, Yao-Nan Wang, Lung-Ming Fu

**Affiliations:** 1 Department of Vehicle Engineering, National Pingtung University of Science and Technology, Pingtung 912, Taiwan; E-Mails: clchang@mail.npust.edu.tw (C.-L.C.); jcleong@mail.npust.edu.tw (J.-C.L.); 2 Department of Materials Engineering, National Pingtung University of Science and Technology, Pingtung 912, Taiwan; E-Mail: tfhong@mail.npust.edu.tw

**Keywords:** capillary electrophoresis microchip, injection system, expansion chamber

## Abstract

This study presents an experimental and numerical investigation on the use of high-resolution injection techniques to deliver sample plugs within a capillary electrophoresis (CE) microchip. The CE microfluidic device was integrated into a U-shaped injection system and an expansion chamber located at the inlet of the separation channel, which can miniize the sample leakage effect and deliver a high-quality sample plug into the separation channel so that the detection performance of the device is enhanced. The proposed 45° U-shaped injection system was investigated using a sample of Rhodamine B dye. Meanwhile, the analysis of the current CE microfluidic chip was studied by considering the separation of Hae III digested ϕx-174 DNA samples. The experimental and numerical results indicate that the included 45° U-shaped injector completely eliminates the sample leakage and an expansion separation channel with an expansion ratio of 2.5 delivers a sample plug with a perfect detection shape and highest concentration intensity, hence enabling an optimal injection and separation performance.

## 1. Introduction

In recent years, integrated microfluidic devices, also known as “Lab-on-a-Chip”, have been utilized for the analysis of chemical or biological assays in biochemistry, biophysics, medicine and life sciences [1–10]. Microfluidic chips offer researchers many potential benefits, including a high sample throughput, integrated sample processing, minimized sample and reagent volume, improved performance and reliability, and integration of the chip and detection circuitry. Now, many functional microfluidic devices have been developed to perform a variety of tasks, including sample pre-treatment and injection, species mixing, polymerase chain reaction, and cell/particle separation and counting [11–21].

The injector and separation channel are the key elements of a CE microfluidic device, which is a cross-shaped microchannel that uses electrokinetic flow to load samples into the intersection of one channel and dispenses a minute quantity of sample into the separation channel. The precise control of the sample injection and separation processes in microfluidic dispensers is very important to the analysis. One of the most common sample injection methods in CE microfluidic devices is the cross-shaped injection technique. The floating sample injection method causes the sample leakage to increase the signal baseline as the number of injection runs increases, thus reducing the separation efficiency [22–27]. Fu *et al.* [22] identified severe sample leakage in the floating sample injection method when high voltage gradients were established. The sample leakage effect was found to increase the signal baseline as the number of injection runs increased resulting in the reduction of the separation efficiency. Consequently, the authors developed a low-leakage injection technique designed specifically to improve the detection performance of microfluidic devices. Zhuang *et al.* [27] developed a novel electrokinetic double-focusing injection technique for a capillary electrophoresis microchip, which involved four accessory arm channels in which symmetrical focusing potentials were loaded to form a unique parallel electric field distribution at the intersection of the injection channel and separation channel. The parallel electric field effectuates virtual walls to confine the spreading of the sample plug at the intersection and prevents sample leakage into the separation channel.

Electrokinetic focusing may be used to create a variable sample plug volume injector that produces consistent sample plug volumes. The improved sample plug distribution in the separation channel was reported to provide an enhanced separation performance [28,29]. Luo *et al.* [30] presented a double-cross hydrostatic pressure sample injection for a capillary electrophoresis microchip. The injection method uses hydrostatic pressure generated by emptying the sample waste reservoir for sample loading and electrokinetic force for dispensing. One cross, created by the sample and separation channels, is used for the formation of a sample plug. Another cross, formed by the sample and controlling channels, is used for plug control. This injection method enables linear adjustment of the sample plug volume and has the ability to reduce the number of electrodes to a minimum for either single- or multi-channel electrophoresis.

Some studies related to the improved sample injection and separation technique in microchip electrophoresis have been performed. For example, Wu and Yang [31] presented a T-form electrokinetic injection system for the discrete time-based loading and dispensing of samples of variable-volume in a microfluidic chip. The push-pull effect, which is produced during the loading and dispensing processes, can shape a compact sample plug and hence improve the detection resolution of the microfluidic device. Wenclawiak and Püschl [32] showed a mini-review of the sample injection for capillary electrophoresis on a micro fabricated device with on-chip CE injection. They introduced different injection designs (including T-type, double T-type, and cross) and different injection modes (such as pinched injection, gated injection, optically gated injection, pressure/pneumatic injection, and double L injection), which were applied to various analytes in advanced studies. In addition, Tsai *et al.* [33–35] presented a microfluidic CE device incorporating a conventional cross-form injection system and an expansion chamber located at the inlet of the separation channel. The geometry of the expansion channel was optimized numerically and the performance of the device was then verified experimentally by carrying out the separation of a mixed *Hae III-digested ϕx-174 DNA sample*.

Despite the notable contributions of the various injection schemes discussed above, research still continues into the development of enhanced sample injection techniques for CE microchips [36–45]. In a recent study, Blas *et al.* [37] presented a comparative study of two electrokinetic injection modes in chips: the floating, which has been mainly used up to now, and the dynamic. It appeared that the dynamic injection mode is much more appropriate than the floating mode as it is faster, more reproducible, and leads to more efficient separation for the same injected amount, whatever the amount, because the sample plug is less dispersed. Lee *et al.* [44] presented a numerical study of the 3-D characteristics of electroosmotic injection and migration of sample species in CE microchip. A non-rectangular cross section resulted in a vertically non-uniform sample plug during the loading stage. Conventional 2-D simulation approach can result in up to 40% errors in the calculation of injection qualities. It was also found that the 2-D simulation using a proper channel width has a good agreement with the 3-D simulation data.

**Figure 1:**
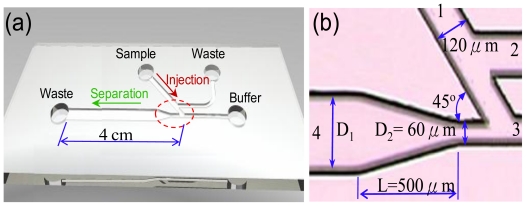
Schematic illustration of proposed microfluidic chip.

To achieve high-performance detection in a CE microfluidic device, the sample leakage effect and shape of the sample plugs separated into the separation channel is of great importance. Therefore, this study presents an integrated CE microfluidic device that combines a 45° U-shaped injection system with an expansion chamber at the inlet of the separation channel to eliminate the sample leakage effect and deliver high-quality sample bands into the separation channel. A schematic illustration and photograph of the current CE microfluidic device are presented in Figure 1. This study shows that the proposed low leakage injector improves the sample separation and the current expansion chamber advances the high-quality sample bands into the separation channel to enhance the detection performance.

## 2. Chip Fabrication and Experimental Setup

The current microfluidic devices were fabricated on microscope glass slides (76 × 26 × 1 mm^3^, Marienfeld, Germany) using standard photolithographic procedures followed by wet chemical etching. Prior to fabrication, the slides were annealed at 400 °C for 4 h to release any internal residual stresses. Initially, microscope slides were cleaned using boiling piranha solution and then patterned with positive photoresist (AZ 4620, Clariant Corp., USA) and UV exposure using a standard photolithography procedure. The patterned glass substrates were then directly immersed in a dilute BOE (6:1), stirred HF/NH_4_F bath for 30 min with ultrasonic agitation to generate the 25 μm deep microchannels. The fluid-via-holes were drilled by a diamond drill-bit on the etched upper plate. A fusion bonding process was then used to achieve high strength bonding results with a high yield. Briefly, this procedure consisted of the following steps: the two glass flats were carefully aligned and made to cling to each other using deionized (DI) water, the two glasses were held together by atmospheric pressure, and the microchip was sintered above 580 °C for 20 min with a ramp rate of 5 °C/min^−1^. A sealed microfluidic device was able to be formed after bonding the two glass plates. Note that the current authors presented a detailed description of the chip fabrication method for wet chemical etching in a previous study [46,47].

The microfluidic chip performance was monitored by laser-induced fluorescence (LIF). The fluid sample manipulations performed in the current study were observed by mercury lamp induced fluorescence using a charge-coupled device (CCD; model DXC-190, SONY, Japan) for imaging purposes. The study used 10^−3^ M sodium borate (pH 8.5 Aldrich) as the buffer fluid, and 10 ^−4^ M Rhodamine B fluorescent dye as the sample. The DNA concentration and electrophoretic separation performance investigations were conducted using a Hae III digested ϕx-174 DNA (Amersham Bioscience, Sweden) sample consisting of eleven fragments (72, 118, 194, 224, 271, 281, 310, 603, 872, 1078 and 1353 base pairs). Detection of the Hae III digested ϕx-174 DNA sample was performed using a CE buffer of 1.2% HPMC (Hydroxypropyl Methyl Cellulose) in TBE (tris-borate-EDTA) with 1% SYBR green I (Molecular probes, USA) fluorescent dye. The experimental images were first captured by an optical microscope (mode E400 Nikon, Japan), then filtered spectrally (550 nm cut-on), and finally measured by a CCD device. The voltage switching apparatus was computer-controlled using code written in-house in Labview. Finally, an APD module (Avalanche Photo-Diode, C5640-01, Hamamatsu, Japan) was used to detect the emitted optical signals.

## 3. Numerical Modeling

Numerical simulation of this problem requires solving for the electric potential, zeta potential, ionic concentration, velocity component, and sample concentration throughout the computational domain. A new mathematical model of the electrokinetic transport phenomena in a microfluidic dispenser is developed here with consideration of the spatial gradient of conductivity. The model considers the processes as two-dimensional, which is very common in the modeling of similar processes. Regarding the numerical simulation of electroosmotic flows, the authors previously developed physical models based on (a) the Poisson equation (Ψ) for the electric potential and zeta potential at the fluid-solid boundary, (b) the Nernst-Planck equations (n^+^, n^−^) for the positive and negative ionic concentrations, (c) the full Navier-Stokes equations (u, v) modified to include the effects of the body force due to the electric and charge density, and (d) a sample concentration equation for the sample plug distribution. Note that the detailed expressions of the governing equation, the initial conditions, and the boundary conditions were presented in previous studies by the authors [48].

## 4. Results and Discussion

In a CE microfluidic device, the shape of the delivered sample plug depends primarily on the electroosmotic flow pattern at the intersection of the channels, and plays an important role in determining the resolution of the electrophoresis analysis. Therefore, the injector and separation chamber is one of the key elements in the handling of the sample and delivering process of the CE micofluidic device.

**Figure 2:**
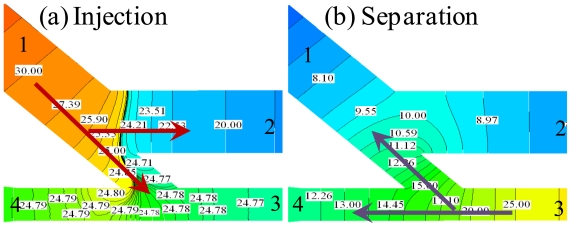
Electrical potential contours in 45° U-shaped injection method: (**a**) injection and (**b**) separation steps with an electric flied of 300 V/cm.

Figure 2 presents the electric potential contours during the injection and separation steps of the 45° U-shaped injection method in the proposed microfluidic device. The figure shows that the injection and separation steps were performed using an electric field intensity of 300 V/cm. During the injection step, the sample was loaded from channel 1 to channel 2 by applying an electric field of 300 V/cm (channel 1 is kept at a potential *Φ*_1_, channel 2 is held at ground potential). Channels 3 and 4 are electrically floating, resulting in a potential below that of *Φ*_1_ and, therefore, sample flow from channel 1 towards the intersection 3/4. Therefore, potential gradients existed in channels 1 → 3 and 1 → 4, and this is called the inertial force (*ρ*(*u.* ∇)*u*, where *ρ* is the density of the sample, *u* is velocity of sample, and ∇ is operator) (Figure 2a). When the sample had completely filled the intersection of channels 3 and 4, the electrical potential switched immediately to the separation step in which channels 1 and 2 were floating (*i.e.*, ∂φ / ∂*x* = 0) and an electric field intensity of 300 V/cm was applied across channels 3 → 4. The sample plug was delivered to separation channel 4, and the sample flow in channel 1 was pulled back by the inertial force from channel 3. Therefore, the sample leakage can be completely eliminated by this proposed 45° U-shaped injector.

**Figure 3:**
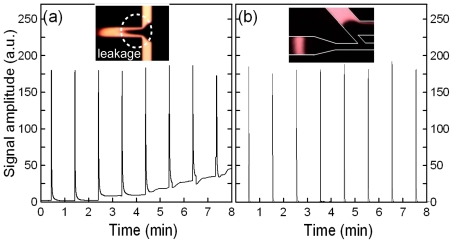
Acquired optical signals for a series of 8 injection/separation cycles performed with applied electric field of 300 V/cm with (**a**) cross-shaped and (**b**) 45° U-shaped injectors, respectively.

To interpret the effects of sample leakage on the resolution sensitivity of this proposed 45° U-shaped injector, a constant electric field of 300 V/cm was established and a series of injection/separation cycles was performed using a test sample of 10^−4^ M Rhodamine B. The sample was excited within the detection region (located 2.5 cm downstream from the intersection of channels 3 and 4) using a mercury lamp, and an APD module was then used to detect the resulting optical signals. Figure 3a,b present the acquired optical signals over a series of 8 injection/separation cycles performed with an electric separation field of 300 V/cm in cross-shaped and 45° U-shaped injectors, respectively. For the traditional cross-shaped injector, the baseline begins to amass as the number of cycles increases (Figure 3a). This drift effect is the result of sample leakage from the side channels, clearly reducing the quality of the detection performance. Figure 3b presents the optical signals acquired over 8 injection/separation cycles performed using a 45° U-shaped injector. Compared with the results presented in Figure 3a, it can be seen that the baseline remains stationary over the 8 cycles. This indicates that the injector entirely eliminates sample leakage and is therefore suitable for applications requiring a high detection resolution. Note that more details of the sample leakage effects were presented in previous studies by the authors [21,23].

**Figure 4:**
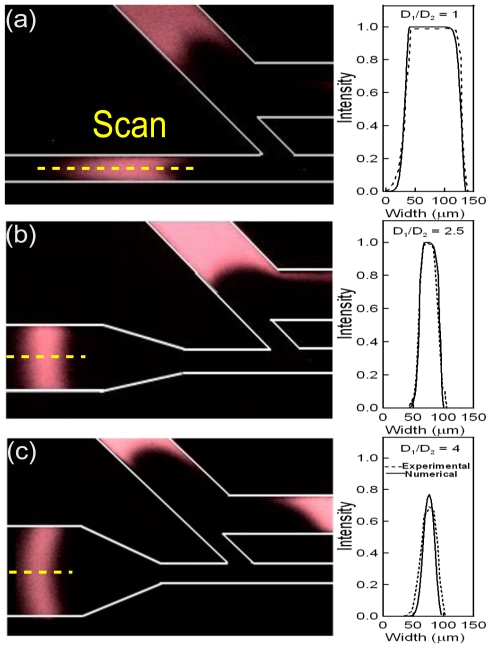
Sample shape in separation processes and concentration profiles at the centerline of the sample band for a constant expansion length of 500 μm and different expansion ratios, *i.e*., (**a**) 1, (**b**) 2.5, and (**c**) 4.

Figure 4 shows the sample plug shape distribution in the separation process and the concentration intensity profiles at the centerline of the sample plug that pass through a constant expansion length (L) of 500 μm with different expansion ratios. The expansion length (L) is set to 500 μm because this results in a very orthogonal and very small variance sample plug in the separation channel. It is suitable for enhancing the resolution sensitivity of detection. Note that more details on the effect of the expansion length were presented in previous studies by the authors [33–35]. In Figure 4, it is clear that the low expansion ratios of 1 and 2.5 still result in a high intensity sample plug concentration being maintained in the separation channel. However, for an expansion ratio of 1 (*i.e*., no expansion effect), Figure 4a indicates that the sample band distribution is rather broad. This reduces the separation efficiency. When the expansion ratio is increased to 2.5 (as shown in Figure 4b), it is clear that the broad sample plug distribution is greatly improved. The concentration intensity remains at its highest value, but the sample plug distribution is significantly narrower. When the expansion ratio is increased to 4 (as shown in Figure 4c), the sample plug is dispersed by a larger expansion ratio in the separation channel. The present results indicate that the concentration intensity reduces by about 25% (compared to Figure 4a) at the detection area due to dispersion.

**Figure 5:**
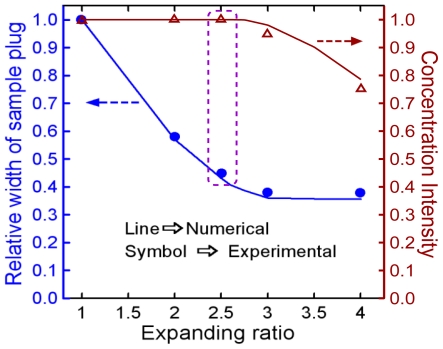
Comparison of concentration intensity and relative width of the sample band for various expanding ratios.

Figure 5 compares the concentration intensity and the relative width of the sample band at a position 1500 μm downstream from the intersection of channels 3 and 4 for different expansion ratios. In this figure, the concentration intensity is calculated by averaging the centerline of five sequential sample plugs. A small concentration intensity indicates that the expansion chamber generates too much dispersion of the sample plug, thereby downgrading the detection performance. Considering the relative width of the sample plug, the maximum sample plug width is obtained at an expansion ratio of 1 (see Figure 4a). In general, a narrow sample plug with high concentration intensity results in a higher theoretical plate number and enhanced separation efficiency. From Figure 5, it is clear that an expansion ratio of 2.5 produces the optimal condition between high concentration intensity and a suitable sample plug width.

**Figure 6:**
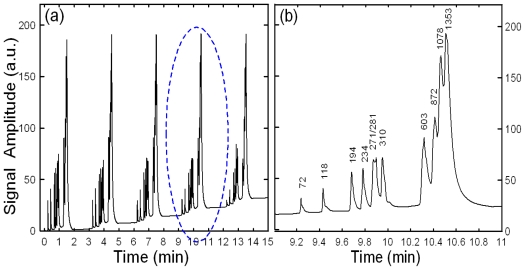
Electropherograms of separated ϕx-174 DNA with conventional cross-shaped separation channels (injection and separation field strength set to 300 V/cm).

Figure 6a presents electropherograms of five consecutive ϕx-174 DNA separations with a conventional cross-channel injection system utilizing an injection/separation field strength of 300 V/cm. The DNA sample was a Hae III digested ϕx-174 DNA consisting of eleven fragments, namely 72, 118, 194, 224, 271, 281, 310, 603, 872, 1078 and 1353 base pairs (bp). In Figure 6a, it can be seen that the baseline begins to drift as the number of injection/separation cycles increases. This phenomenon reflects the effects of sample leakage from injection channels into the separation channel and leads to a significant degradation of the detection resolution. For this sample, eleven distinct peaks were detected in the separation channel. Nevertheless, in the present case, it can be seen that the resolution performance is not desirable, as shown in Figure 6b.

**Figure 7:**
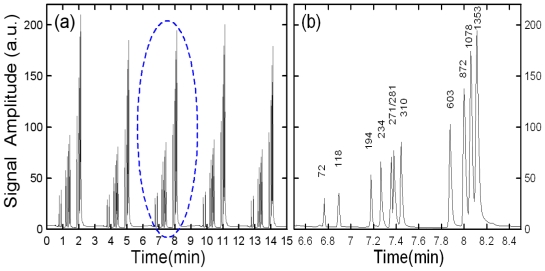
Electropherogram of separation results for ϕx-174 DNA sample using 45° U-shaped injector with expansion chamber of expansion ratio 2.5 (injection and separation voltage of 300 V/cm).

Figure 7a presents five sequential ϕx-174 DNA electropherograms when the same sample is separated with this particular 45° U-shaped injector and expansion chamber (expansion ratio 2.5) with an injection/separation voltage of 300 V/cm. For this sample, the eleven distinct peaks are clearly resolved as shown in Figure 7b. The results clearly demonstrate that the proposed 45° injection structure yields improved separation performance.

## 5. Conclusions

This study proposes a CE microfluidic device with a novel injection structure with a 45° U-shaped injector and an expansion chamber at the inlet of the separation channel to improve the sample plug distribution in the separation step and enhance separation efficiency. This study performed the experimental separation of samples consisting of Rhodamine B fluorescent dye and Hae III digested ϕx-174 DNA samples to explore the effects of the 45° U-shaped injector and expansion chamber on the sample leakage and separation performance. The numerical and experimental results show that the 45° U-shaped injector can efficaciously avoid sample leakage into the separation channel, and that an expansion chamber with an expansion ratio over than 2.5 results in a higher separation efficiency, as the prolongation of the peaks is reduced. A novel 45° U-shaped injection system combined with an expansion chamber at the inlet of the separation channel with an expansion ratio of 2.5 was developed and its performance analyzed. The results indicate that this CE microfluidic device significantly prevents sample leakage and transports suitable sample plugs into the separation channel enhancing the detection performance.
